# 

**Published:** 1896-02

**Authors:** 


### CONTENTS.

ORIGINAL COMMUNICATIONS—

THE TREATMENT OF CUTANEOUS CANCERS. By A. R. Robinson, M.D., New York .... 713

PENETRATING WOUNDS OF THE ABDOMEN. By Randolph Winslow, M.D., Baltimore. 718

A CASE OF SYMMETRICAL GANGRENE (RAYNAUD'S DISEASE), WITH SOME UNUSUAL FEATURES IN ITS ETIOLOGY. By M. B. Hutchins, M.D., Atlanta, Ga............... 722

LARYNGEAL STENOSIS. By W. Jay Bell, A.B., M.D., Atlanta, Ga..................... 724

REPORT OF CLINICS AT ATLANTA MEDICAL COLLEGE. By Ralcey W. Bell............ 726

SOCIETY REPORTS—

ATLANTA SOCIETY OF MEDICINE.......................................... 729

CORRESPONDENCE—

REMOVAL OF MESOBLASTIC TUMOR OF THE NECK.................................. 738

EDITORIAL—

A "QUALIFYDE" PRACTITIONER OF FLOYD COUNTY ON BOARDS, COLLEGES, ETC...... 740

DR. JAMES E. REEVES.................................................. 741

BOGUS MEDICAL JOURNALISM............................................ 742

AN EXPLANATION ABOUT PREMIUMS........................................ 743

SELECTIONS AND ABSTRACTS—

EYE, EAR, NOSE AND THROAT............................................ 744

SURGERY.............................................................. 747

GENERAL MEDICINE AND THERAPEUTICS.................................... 751

OBSTETRICS AND GYNECOLOGY........................................... 763

MISCELLANEOUS........................................................ 767

MEDICAL ITEMS........................................................ 769

BOOK REVIEWS......................................................... 775

ADVERTISERS' INDEX TO ADVERTISEMENTS................................... xiv

MEDICAL ITEMS—CONTINUED.......................................... x, xi, xii, xiii

CLASSIFIED INDEX TO ADVERTISEMENTS.....................................xxxii

